# Dexmedetomidine’s inhibitory effects on acetylcholine release from cholinergic nerves in guinea pig trachea: a mechanism that accounts for its clinical benefit during airway irritation

**DOI:** 10.1186/s12871-017-0345-z

**Published:** 2017-03-29

**Authors:** Maya Mikami, Yi Zhang, Benjamin Kim, Tilla S. Worgall, Harald Groeben, Charles W. Emala

**Affiliations:** 1 Department of Anesthesiology, College of Physicians and Surgeons of Columbia University, 630 West 168th Street, P&S Box 46, New York, NY 10032 USA; 2 Department of Pathology and Cell Biology, College of Physicians and Surgeons of Columbia University, New York, NY USA; 3 Department of Anesthesiology, Intensive Care and Pain Therapy, Kliniken Essen-Mitte, Essen, Germany

**Keywords:** Airway Management, α2 adrenoceptor agonist, Anesthetic Agents, Bronchodilation, Muscle Relaxation, Smooth Muscle

## Abstract

**Background:**

Airway instrumentation can evoke upper airway reflexes including bronchoconstriction and cough which can cause serious complications including airway trauma, laryngospasm or bronchospasm which may in turn lead to difficulty with ventilation and hypoxemia. These airway events are mediated in part by irritant-induced neuronal modulation of airway tone and cough responses. We investigated whether the commonly used anesthetic agents dexmedetomidine, lidocaine or remifentanil attenuated neuronal and airway smooth muscle responses in the upper airways of guinea pigs.

**Methods:**

The ability of dexmedetomidine, lidocaine or remifentanil to attenuate direct cholinergic nerve stimulation, C-fiber stimulation or direct smooth muscle contraction were studied using isolated tracheal rings from male guinea pigs under four paradigms; (1) the magnitude of contractile force elicited by cholinergic electrical field stimulation (EFS); (2) the amount of acetylcholine released during cholinergic EFS; (3) the direct airway smooth muscle relaxation of a sustained acetylcholine-induced contraction and (4) the magnitude of C-fiber mediated contraction.

**Results:**

Dexmedetomidine (1–100 μM) and lidocaine (1 mM) attenuated cholinergic 30Hz EFS-induced tracheal ring contraction while remifentanil (10 μM) had no effect. Dexmedetomidine at 10 μM (*p* = 0.0047) and 100 μM (*p* = 0.01) reduced cholinergic EFS-induced acetylcholine release while lidocaine (10 μM-1 mM) and remifentanil (0.1–10 μM) did not. Tracheal ring muscle force induced by the exogenous addition of the contractile agonist acetylcholine or by a prototypical C-fiber analogue of capsaicin were also attenuated by 100 μM dexmedetomidine (*p* = 0.0061 and *p* = 0.01, respectively). The actual tracheal tissue concentrations of dexmedetomidine achieved (0.54–26 nM) following buffer application of 1–100 μM of dexmedetomidine were within the range of clinically achieved plasma concentrations (12 nM).

**Conclusions:**

The α2 adrenoceptor agonist dexmedetomidine reduced cholinergic EFS-induced contractions and acetylcholine release consistent with the presence of inhibitory α2 adrenoceptors on the prejunctional side of the postganglionic cholinergic nerve-smooth muscle junction. Dexmedetomidine also attenuated both exogenous acetylcholine-induced contraction and C-fiber mediated contraction, suggesting a direct airway smooth muscle effect and an underlying mechanism for cough suppression, respectively.

## Background

Cough and bronchoconstriction are airway reflexes that provide defense and protection for the respiratory tract from inspired noxious agents and are thus a common response to an airway foreign body such as an endotracheal tube or suction catheter. However, during induction and emergence from general anesthesia and extubation in the intensive care unit, these protective airway reflexes can cause serious complications, including airway trauma, increased intracranial, intra-abdominal or intraocular pressures, surgical bleeding, wound dehiscence, cardiovascular instability, laryngospasm and/or bronchospasm [1]. Moreover, in the intensive care setting, obstructive airway diseases like asthma and chronic obstructive pulmonary disease (COPD) can complicate weaning from mechanical ventilation and lead to weaning failures and multiple weaning attempts [2]. These airway events occur in part by irritant-induced neuronal modulation of airway reflexes and airway tone [3]. Activated afferent sensory fibers originating in the upper airway synapse in the nucleus of the solitary tract which projects excitatory glutamatergic and inhibitory γ-aminobutyric acid-A (GABA_A_)-ergic neurons to the airway-related vagal preganglionic neurons (AVPN). Parasympathetic nerves originate from the AVPN, and after synapsing at parasympathetic ganglia release acetylcholine from post-ganglionic cholinergic nerves. The release of acetylcholine is dysregulated in some animal models of asthma [4] and possibly in human asthmatics [5], suggesting a mechanistic basis for clinically observed reflex-induced bronchoconstriction following airway manipulation. By modulating receptors on the pre-junctional side of postganglionic parasympathetic nerves, acetylcholine release can be attenuated. These receptors include M2 muscarinic, mu opioid [6], GABA_B_ [7], β2 adrenoceptor and α2 adrenoceptors [8].

Dexmedetomidine is a highly selective and potent α2 adrenoceptor agonist [9], and is known to produce both hypnotic-anesthetic effects and analgesia in animal models [10, 11] and humans [12, 13]. The drug causes cooperative conscious sedation by stimulating the locus coeruleus in the brain stem resulting in inhibition of the sympathetic vasomotor center of the brain as opposed to other sedatives (e.g. propofol, benzodiazepines) which act upon GABAergic pathways causing unconsciousness. Unlike other sedatives, dexmedetomidine does not cause significant respiratory depression in clinically relevant doses in either healthy volunteers [14] or surgical patients who require intensive care [15]. Intraoperative infusions of dexmedetomidine reduce the requirement for perioperative opioids [16], and reduce the hemodynamic response to intubation and extubation [17]. These advantages of dexmedetomidine, along with a decrease in saliva production, make dexmedetomidine an attractive choice for sedation during awake fiberoptic intubation [18] or for smooth extubation in both the operating room and intensive care unit.

Many other medications have been utilized to optimize the conditions for awake fiberoptic intubation including topical and/or intravenous local anesthetics, opioids and benzodiazepines. The clinical utility of these medications in reducing airway responses during awake intubation or emergence from anesthesia may also be mechanistically explained by activation of pre-junctional receptors on postganglionic parasympathetic nerves leading to decreased acetylcholine release. We questioned whether dexmedetomidine, lidocaine or remifentanil attenuated acetylcholine release from electrically field stimulated nerves in guinea pig trachea and whether they attenuated airway smooth muscle contraction induced by electrical field stimulation (EFS), C fiber activation or exogenous acetylcholine.

## Methods

### Guinea pig tracheal rings in organ baths

Studies were approved by the Columbia University Animal Care and Use Committee (protocol number AC-AAAH4701). Tracheal rings were isolated from male Hartley guinea pigs under pentobarbital anesthesia. Connective tissue and epithelium were removed and tracheal rings were suspended in 4 ml water-jacketed organ baths as previously described [19]. Organ baths contained Krebs-Henseleit (KH) buffer of the following composition: (in mM): NaCl 118, KCl 5.6, CaCl_2_ 0.5, MgSO_4_ 0.2, NaHCO_3_ 25, NaH_2_PO_4_ 1.3, D-glucose 5.6, (pH 7.4) with 10 μM indomethacin. The buffer was maintained at 37 °C and continuously bubbled with 95% O_2_/5% CO_2_. The effects of the anesthetic agents dexmedetomidine, lidocaine and remifentanil were then studied under four paradigms in the absence or presence of these anesthetics; (1) the magnitude of the contractile force induced by cholinergic EFS; (2) the amount of acetylcholine released into the buffer during cholinergic EFS stimulation; (3) the relaxation of a sustained exogenous acetylcholine-induced contraction; and (4) the magnitude of the contractile force induced by exogenous capsaicin which activates transient receptor potential cation channel subfamily V member 1 (TRPV1) receptors on C fibers which releases substance P to cause airway smooth muscle contraction.

In those experiments in which acetylcholine released into the buffer was measured during cholinergic EFS, the buffer was further supplemented with 0.1 μM atropine, 10 μM guanethidine and 0.1 μM neostigmine. Atropine was added to block presynaptic inhibitory muscarinic receptors on peripheral cholinergic nerves to maintain stimulus-evoked release of acetylcholine [20]. Neostigmine was used to prevent the degradation of released acetylcholine. Preliminary studies were performed to determine the optimal concentration of neostigmine in the organ bath buffer that protected released acetylcholine from degradation without inhibiting the subsequent acetylcholine detection assay. Guanethidine was included in the buffer to prevent the release of norepinephrine from the sympathetic nerves within the tracheal rings in response to EFS, since catecholamines can modify airway cholinergic activity [21, 22].

### Airway smooth muscle contractile response induced by cholinergic electrical field stimulation

After equilibration of the guinea pig tracheal rings for 100 mins at a resting tension of 1 g with a change in the buffer solution every 15 min, cholinergic EFS was delivered as previously described [19]. In brief, a Grass S48 stimulator (Grass Instrument/Natus Neurology, Warwick, RI) was used with Med-Lab Stimu-Splitter II (Natus Neurology) to deliver DC current (30 Hz, 24 V, 0.5 ms pulse width in single 5 s trains, 0.013 trains/s) to tracheal rings. Following stable EFS-induced contractions, 10 μM remifentanil, 100 μM dexmedetomidine, or 1 mM lidocaine were added to the buffer with the continuous measurement of the contractile force elicited by EFS. In a separate set of experiments, cumulative concentrations of dexmedetomidine (0.1–100 μM) were added to the buffer during cholinergic EFS-induced contractions.

### Acetylcholine release induced by EFS

In separate experiments, after equilibration of the tracheal rings under 1 g resting tension for 100 mins, 0.1 μM atropine, 10 μM guanethidine and 0.1 μM neostigmine were added to the buffer for 30 min, followed by the application of cholinergic EFS (2 Hz) for 15 min at which time the organ bath buffer was collected. The organ bath was again filled with new buffer with or without cumulatively increasing concentrations of remifentanil (0.1–10 μM), lidocaine (10 μM-1 mM), dexmedetomidine (1–100 μM) or tetrodotoxin (1 μM). Tracheal rings were incubated with each concentration of the above test drugs for 30 min before each 15 min EFS followed by collection of the organ bath buffer for measurements of released acetylcholine. EFS was applied to all the tracheal rings for four 15 min periods, and one tracheal ring received only the drug vehicle and served as a time control in each experiment. Each of the collected aliquots of organ bath buffer (4 ml) were immediately frozen at -80C. Frozen buffer samples were lyophilized (FreeZone 2.5 Plus, Labconco, Kansas City, MO) and reconstituted with 500 μl water. Acetylcholine was measured using a choline/acetylcholine quantitation kit (Sigma-Aldrich, St. Louis, MO). Measured acetylcholine were expressed as a percentage of that measured in the first sample (first EFS) of each bath. EFS frequency was chosen based on our previous experience [19] and published literature [23] to optimize acetylcholine release.

### Airway smooth muscle contractile response induced by exogenous acetylcholine and capsaicin

Tracheal rings were contracted with cumulatively increasing concentrations of acetylcholine (0.1–100 μM) twice, washed six times until resting tension was re-established at 1 g, then contracted again with an approximate EC_50_ concentration of acetylcholine determined for each tracheal ring (1–1.5 μM). After the achievement of a plateau in the induced contractile force, dexmedetomidine (0.1–100 μM) was added to the baths to measure the relaxation of acetylcholine pre-contracted tracheal rings.

In separate experiments, tracheal rings were contracted with cumulatively increasing concentrations of acetylcholine (0.1–100 μM) twice, washed six times until resting tension was re-established at 1 g, then pretreated with nothing, remifentanil (0.1–10 μM), lidocaine (10 μM–1 mM), dexmedetomidine (1–100 μM) or 1 μM tetrodotoxin for 20 min before the addition of 10 μM N-vanillylnonanamide (synthetic analogue of capsaicin) which activates TRPV1 receptors on C fibers in this preparation to release substance P resulting in smooth muscle contraction. The magnitude of the capsaicin-induced contraction was normalized to the initial maximal acetylcholine contractile force for each individual tracheal ring.

### Tracheal ring tissue dexmedetomidine concentration measurement by mass spectrometry

After equilibration of the tracheal rings under 1 g resting tension for 100 mins, dexmedetomidine (1–100 μM) was added to the buffer for 30 mins, then the rings were briefly rinsed in phosphate buffered saline (PBS) and blotted dry to measure tissue weight. The tissues were homogenized in PBS, centrifuged at 13,000 × g for 10 mins, and the supernatant was collected and protein concentration was measured using the BCA Protein Assay Kit (Pierce, Thermo Fisher Scientific, Waltham, MA). Dexmedetomidine (m/z 201.1.95.1) was quantified by HPLC – MS/MS after extraction with a 1:15 v/v solution of dichloromethane: methanol (1:1) containing sphingomyelin C12 (d18:1/12:0, Avanti Polar lipids) as an internal standard. Sixty μl of sample with known protein concentrations and an external standard of dexmedetomidine (range 0.78–100 μM) were extracted by vortexing at room temperature overnight. After centrifugation to precipitate resulting residues, 4 μl were injected into an Agilent 1200 HPLC (Agilent Poroshell 120) linked to an Agilent 6430 triple quadrupole-tandem mass spectrometry (MS/MS). Mobile phase A was methanol/water/chloroform/formic acid (55:40:5:0.1 v/v); Mobile phase B was methanol/acetonitrile/chloroform/formic acid (48:48:4:0.1 v/v). After equilibration for 0.8 min at 95% mobile phase A, the gradient was gradually increased to 95% mobile phase B that was held for 5 min, and then was finally returned to 95% mobile phase A to re-equilibrate for 2.35 min before the next injection. The flow rate was 0.6 ml/min. The total run duration was 9.25 min. All measurements were performed in triplicate and were averaged. Agilent Mass Hunter Software (QQQ) was used for quantitative analysis. Results were expressed as pmol/mg protein, then converted to pmol/mg tissue and finally to molar units since 1 g of tracheal ring tissue was approximately 1 ml in volume.

### Statistical analysis

Statistical analysis were performed using GraphPad Prism 4.03 software (GraphPad Software, Inc., La Jolla, CA). With the small sample size, non-parametric Mann–Whitney analysis was performed to compare control and each drug tested and *p* < 0.05 was considered statistically significant. For comparisons with the same control group (the released acetylcholine measurements and acetylcholine-induced contractions with remifentanil and lidocaine), adjustments on the significance level was done for the multiple concentration points and *p* < 0.01 was considered statistically significant. We presented results as means and standard error of the mean. Sample sizes were determined using two-sided 5% type I error and a power of 80%. Based on our extensive experience with the guinea pig wire myograph model, a 50% reduction of muscle contractile force was considered significant with a 30% standard deviation. Therefore, *n* = 6 guinea pigs were needed for each wire myograph study. In the study where released acetylcholine was measured, a reduction of the release to 60% was considered biologically significant, and an average of *n* = 7 guinea pigs were needed with a 25% standard deviation.

## Results

**Fig. 1:**
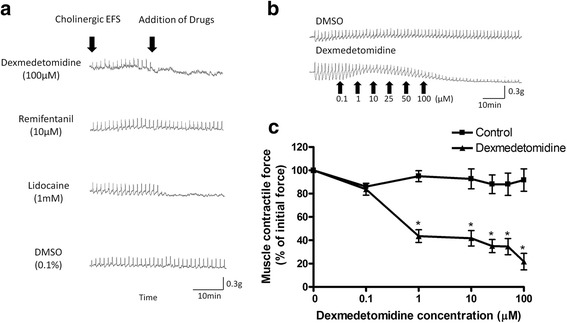
Guinea pig tracheal ring muscle force generated in response to cholinergic electrical field stimulation (EFS). **a** Following stable EFS-induced contractions, 100 μM of dexmedetomidine, 10 μM remifentanil or 1 mM lidocaine or vehicle (0.1% DMSO) were added to buffer solution. Dexmedetomidine and lidocaine inhibited EFS-induced contractions. **b**, **c** Dexmedetomidine attenuates guinea pig tracheal ring muscle force generated in response to cholinergic EFS contraction. Representative trace of EFS-induced muscle force over time with increasing concentrations of dexmedetomidine (**b**) and expressed as percent change from the initial baseline EFS-induced contraction (**c**). Control tracheal rings treated with the vehicle DMSO (0.1%) serves as a time control. Mean ± SEM (*n* = 4). *p < 0.05

**Fig. 2:**
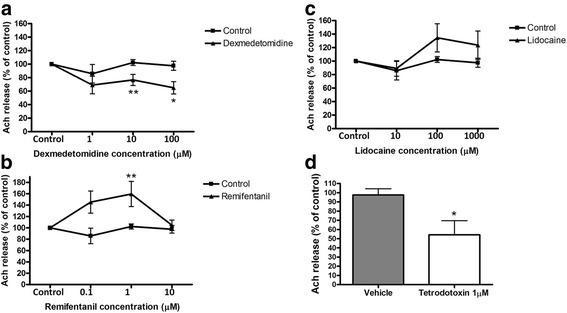
Effect of dexmedetomidine (**a**), remifentanil (**b**), lidocaine (**c**) and tetrodotoxin (**d**) on acetylcholine (Ach) release from guinea pig tracheal rings in response to cholinergic EFS. Data were normalized to an initial measurement of Ach release obtained before addition of drugs. Mean ± SEM (*n* = 7). *p < 0.05, **p < 0.01 significant difference from control

Dexmedetomidine (1–100 μM) and lidocaine (1 mM) inhibited 30Hz EFS-induced cholinergic contractions in guinea pig airway smooth muscle while remifentanil (10 μM) had no effect (Fig. 1). Mean differences between control and dexmedetomidine treatment were 51.4% at 1 μM (95.0 ± 4.72 vs 43.6 ± 5.44%) (*p* = 0.04), 50.9% at 10 μM (92.7 ± 8.54 vs 41.8 ± 6.57%) (*p* = 0.04), 53.0% at 25 μM (88.0 ± 7.98 vs 35.0 ± 5.57%) (*p* = 0.04), 53.4% at 50 μM (88.0 ± 9.62 vs 34.6 ± 6.96%) (*p* = 0.04) and 70.0% at 100 μM (91.7 ± 9.59 vs 21.7 ± 7.06%) (*p* = 0.04). Remifentanil attenuated EFS-induced cholinergic contractions when the EFS frequency was lowered to 2Hz, and this inhibition was reversed by adding 500 nM naloxone to the organ bath buffer (data not shown). In an effort to distinguish between neural release of acetylcholine versus direct inhibition of airway smooth muscle contraction, we measured acetylcholine liberated from the tracheal rings into the organ bath buffer. Dexmedetomidine (10–100 μM) attenuated EFS-induced acetylcholine release while remifentanil (0.1–10 μM) and lidocaine (10 μM–1 mM) did not (Fig. 2). The differences between control and dexmedetomidine at 10 μM were 25.4% (102 ± 4.47 vs 76.6 ± 8.29%; *p* = 0.0047) and 32.5% at 100 μM (97.5 ± 6.76 vs 65.0 ± 9.15%; *p* = 0.01). Tetrodotoxin, which is a sodium channel inhibitor known to abolish neural release of acetylcholine [24], decreased acetylcholine release and served as a positive control. Tetrodotoxin (1 μM) showed a mean difference of 43.1% (97.5 ± 6.76 vs 54.4 ± 15.1%; *p* = 0.04).

**Fig. 3:**
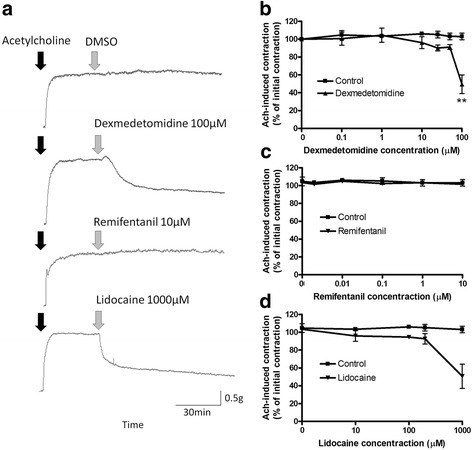
Relaxant effect of dexmedetomidine and lidocaine on exogenous acetylcholine (Ach)-induced contraction of guinea pig tracheal rings. **a** Representative trace of Ach-induced contraction and subsequent relaxation or sustained contraction. **b**, **c**, **d** Following stable Ach-induced contractions, 0.1–100 μM of dexmedetomidine, 2 nM–10 μM of remifentanil, 10–1000 μM of lidocaine or vehicle control (DMSO or water) was added to the buffer solutions and muscle force change was measured. Mean ± SEM (*n* = 5). **p < 0.01

To further distinguish between neural and direct airway smooth muscle mechanisms in attenuating dexmedetomidine’s effect on airway smooth muscle contraction, we contracted the airway smooth muscle with exogenous acetylcholine added to the buffer (independent of neural release) and attempted to directly relax an established contraction with dexmedetomidine. Dexmedetomidine (100 μM) attenuated contractile muscle force induced by the exogenous addition of the contractile agonist acetylcholine while remifentanil did not (Fig. 3). The mean difference between control and dexmedetomidine at 100 μM was 53.4% (103 ± 3.65 vs 49.6 ± 10.5%; *p* = 0.0061). The exogenouse acetylcholine-induced contractile muscle force was 52.3% after 1000 μM lidocaine, but after statistical adjustment for multiple concentration points, this failed to reach statistical significance (103 ± 3.69 vs 50.7 ± 13.6%; *p* = 0.03).

**Fig. 4:**
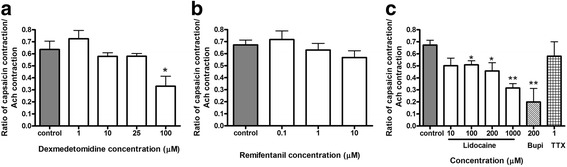
Reduction of capsaicin-induced contraction in guinea pig tracheal rings. Tracheal rings were pretreated with 1–100 μM dexmedetomidine, 0.1–10 μM remifentanil, 10–1000 μM lidocaine, 200 μM bupivacaine or 1 μM tetrodotoxin for 20 min before contraction with 10 μM capsaicin. Dexmedetomidine at 100 μM significantly reduced this contraction (**a**) but remifentanil had no effect (**b**). Local anesthetics lidocaine and bupivacaine (Bupi) attenuated this C-fiber mediated contraction, which are mediated by tetrodotoxin (TTX) –resistant sodium channels. Mean ± SEM (*n* = 6). *p < 0.05, **p < 0.01

In addition to parasympathetic cholinergic fibers traveling in the vagus nerve, another class of nerves (C-fibers) known as non-adrenergic non-cholinergic (NANC) nerves also participate in afferent and efferent irritant reflexes in the upper airway and trachea. A synthetic analogue of capsaicin, N-vanillylnonanamide, a prototypical C-fiber stimulus in the somatosensory system in mammalian lungs, was used to induce TRPV1 receptor-mediated C-fiber activation [25]. In these tracheal rings the activation of C-fibers releases substance P which acts on neurokinin receptors on the airway smooth muscle to induce contraction. As expected, lidocaine and another local anesthetic, bupivacaine, decreased capsaicin-induced contractions, and this effect was mediated by tetrodotoxin insensitive sodium channels since tetrodotoxin at 1 μM had no effect. Dexmedetomidine at 100 μM also significantly decreased this contraction (expressed as a decrease in the ratio of the capsacin/acetylcholine-induced contractions) (mean difference 0.305 (control capsaicin/acetylcholine contraction − 100 μM dexmedetomidine capsaicin/acetylcholine contraction); 0.636 ± 0.0698 vs 0.331 ± 0.0829; *p* = 0.01) while remifentanil had no effect (Fig. 4). The mean differences in the capsaicin/acetylcholine contraction ratios between control and lidocaine at 100 μM were 0.165 (0.673 ± 0.0399 vs 0.508 ± 0.0346; *p* = 0.02), 0.215 at 200 μM (0.673 ± 0.0399 vs 0.458 ± 0.0676; *p* = 0.03) and 0.357 at 1000 μM (0.673 ± 0.0399 vs 0.316 ± 0.0365; *p* = 0.002). The mean difference in the capsaicin/acetylcholine contraction ratio between control and bupivacaine at 200 μM was 0.473 (0.673 ± 0.0399 vs 0.200 ± 0.112; *p* = 0.001).

In the current experiments, dexmedetomidine (0.1–100 μM) was added to an external buffer bathing a segment of airway tissue. The actual achieved tissue concentrations of dexmedetomidine at the site of action (nerve or smooth muscle) is dependent on many factors that determine passive tissue penetration. We sought to determine what the actual average tissue concentration of dexmedetomidine was at 30 min after buffer addition in order to more practically relate the average tissue concentrations to plasma concentrations achieved during clinical use. The measured tissue dexmedetomidine concentrations are shown in Table 1. For buffer concentrations of 1, 10 and 100 μM dexemedetomidine, the average tissue concentration ranged from 0.54 to 26 nM which is within the clinically achieved plasma concentrations (~5 nM) achieved with dexmedetomide infusions.

**Table 1:** Dexmedetomidine concentration

Added in organ bath buffer (μM)	1	10	100
Tracheal ring tissue, Mean ± SEM (nM)	0.54 ± 0.082	3.6 ± 0.39	26 ± 3.9

Table 1 tissue concentrations of dexmedetomidine in guinea pig tracheal rings (*n* = 5 animals). Guinea pig tracheal rings were incubated for 30 mins in buffer with 1–100 μM dexmedetomidine. Following extraction, average penetrating tissue concentrations of dexmedetomidine were deteremined by HPLC – MS/MS

## Discussion

In the operating room, clinicians frequently encounter a patient where intubation of the trachea needs to occur while maintaining spontaneous ventilation and while avoiding agitation, stress, cough and bronchoconstriction. The same type of patient may also require a smooth extubation in the operating room or a controlled ventilator weaning process in the intensive care unit. Failure to successfully wean from mechanical ventilation can lead to days of additional requirement for mechanical ventilatory support with its associated morbidities such as ventilator associated pneumonia. Various medications have been utilized to achieve a smooth awake intubation or extubation which have been particularly focused on the avoidance of cough and bronchoconstriction, which may lead to serious complications such as hypoxemia and airway trauma. Cough and bronchoconstriction are natural airway protective reflexes that are mediated by the same type of irritant receptors resulting in the activation of separate afferent and efferent neural pathways [26]. In the current study we questioned whether clinically used anesthetic agents modulate upper airway responses by modulating cholinergic or C-fiber neural activity and/or have a direct relaxant effect on airway smooth muscle. Although these agents may also contribute to airway tone, or alter the hypercapnic ventilatory response [14] and hypoxic control of breathing [27] in vivo through central effects at the level of the brain or spinal cord, we utilized an ex vivo model in order to assess the peripheral airway effects of these agents. The primary findings were that dexmedetomidine was most potent at inhibiting cholinergic nerve activation, a peripheral parasympathetic neural pathway well appreciated to mediate reflex-induced bronchoconstriction via the release of acetylcholine.

**Fig. 5:**
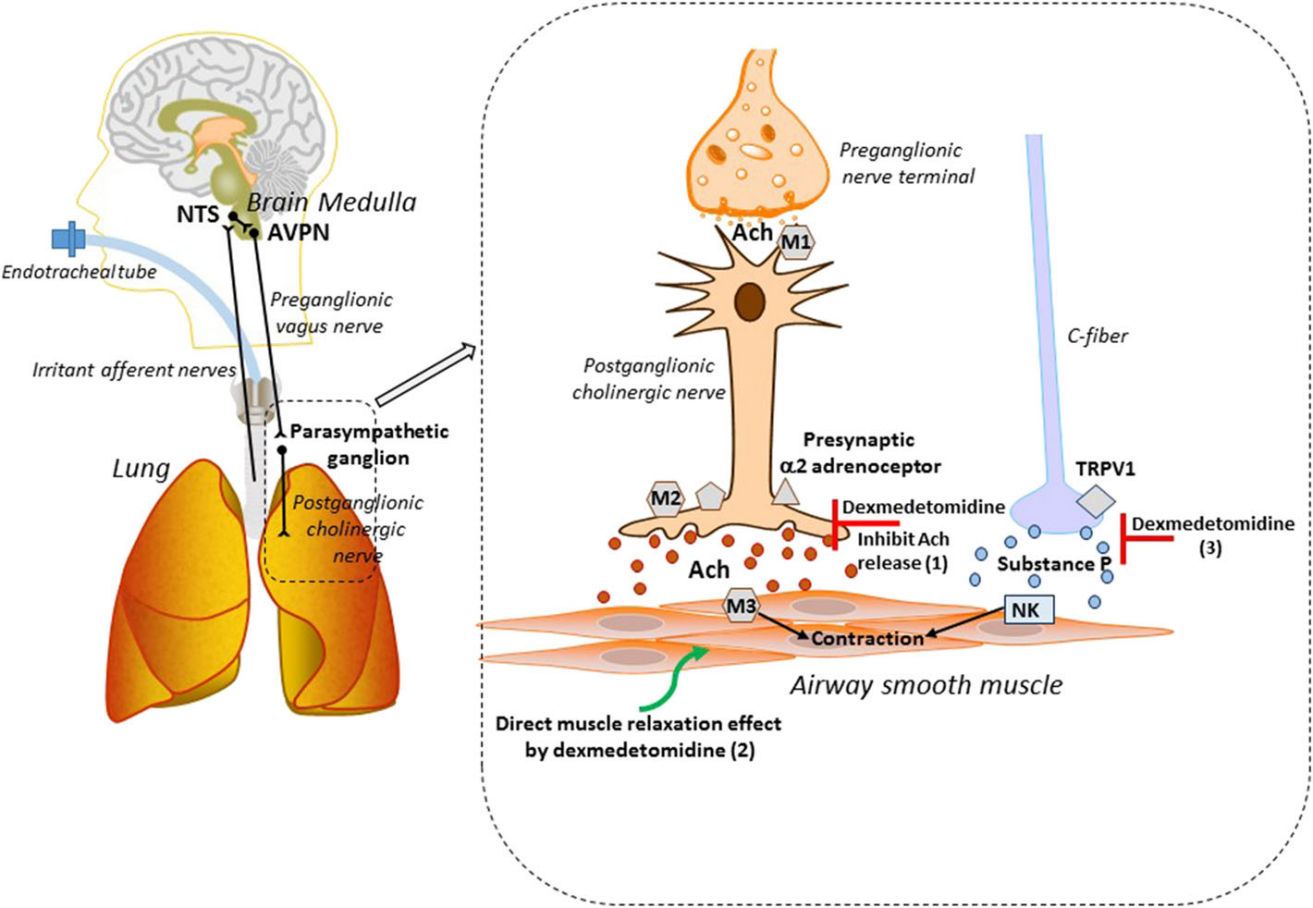
Schematic diagram of dexmedetomidine’s effect on airway smooth muscle (ASM) contractile tone. Sensory information from the upper airway (e.g. irritation by endotracheal tubes or suction catheters) reaches the brain medulla (nucleus tractus solitarius (NTS)) via afferent sensory pathways. Signaling in the NTS is mediated by neurotransmitters including γ-aminobutyric acid (GABA), glutamate and neurokinins, and sends projections to the airway-related vagal preganglionic neurons (AVPN). These neurons are the origin of the parasympathetic nerves, which, after synapsing at peripheral parasympathetic ganglia, release acetylcholine (Ach) from post-ganglionic cholinergic nerves. Released Ach binds to muscarinic receptors (M3 on ASM) and causes ASM contraction. The ganglionic M1 muscarinic receptor is known to facilitate neurotransmission, and neuronal presynaptic M2 muscarinic receptors are known to inhibit Ach release. C-fiber efferents express TRPV1 (transient receptor potential family vanilloid 1) which is a receptor for capsaicin, which induces the release of substance P which binds to the neurokinin (NK) receptors and also facilitates ASM contraction. In the current study, we demonstrated that: (1) dexmedetomidine inhibits Ach release from postganglionic cholinergic nerves; (2) dexmedetomidine directly relaxes an Ach-induced ASM contraction; and (3) dexmedetomidine attenuates C-fiber mediated contraction

A large number of clinical studies support the utility of the α2 adrenoceptor agonist dexmedetomidine in patients who require smooth intubation or extubation of the trachea [28–30] or who require sedation during prolonged ventilator support. In mechanically ventilated patients, dexmedetomidine was reported to improve respiratory mechanics by increasing compliance [31]. Previous in vivo animal studies showed that dexmedetomidine prevented histamine mediated bronchoconstriction in dogs using high-resolution computed tomographic imaging [32] and acetylcholine-induced increases in total lung resistance was blocked by dexmedetomidine in guinea pigs [33]. In vitro, dexmedetomidine was shown to block carbachol-induced smooth muscle tension in guinea pig tracheal ring preparations [33] and methacholine-induced contraction in rats [34], suggesting that dexmedetomidine may have a direct inhibitory effect on airway smooth muscle contraction in the absence of neural stimulation. Although the effect of α2 adrenoceptor agonists on EFS-induced airway smooth muscle contraction has been studied [22, 35], these previous studies only measured a change in smooth muscle contractile force which does not distinguish between a pre-junctional neural effect versus a post-junctional airway smooth muscle effect. In contrast, in the current study we have directly measured acetylcholine release as well as smooth muscle contractile force under both neural stimulation of acetylcholine release or the exogenous addition of acetylcholine, in the presence of several clinically used anesthetic agents commonly chosen during awake instrumentation of the upper airway. We found that the α2 adrenoceptor agonist dexmedetomidine (10 μM) was most potent at reducing cholinergic EFS-induced acetylcholine release (see Fig. 5 for summary) and resultant smooth muscle contraction in guinea pig trachea consistent with the presence of α2 adrenoceptors on the prejunctional side of the postganglionic parasympathetic nerve [22].

At higher concentrations (100 μM) dexmedetomidine directly relaxed airway smooth muscle that had been contracted with acetylcholine (see Fig. 5 for summary), suggesting that dexmedetomidine’s clinical effect on attenuating airway responses may not be limited to its effect on presynaptic neurotransmission. In other words, dexmedetomidine not only provides inhibition of reflex bronchoconstriction, but also has potential as an effective bronchodilator in patients with active bronchoconstriction. Our findings are consistent with previous studies demonstrating dexmedetomidine’s direct inhibitory effects on carbachol-induced guinea pig airway smooth muscle contraction [33], and inhibition of methacholine-induced contraction in rats [34]. α2 adrenoceptor mediated calcium signaling has been studied in the cardiovascular system using isolated rat aorta [36] and is responsible for dexmedetomidine-induced vascular smooth muscle contraction via peripheral α-2B adrenoceptor activation, however, the potential effect of dexmedetomidine on calcium signaling in airway smooth muscle remains to be elucidated.

Dexmedetomidine (100 μM) also reduced capsaicin-induced airway smooth muscle contraction, suggesting an effect on efferent C-fiber mediated release of tachykinins (see Fig. 5 for summary). Further studies measuring substance P and/or neurokinin A release would confirm the mechanism for this finding. Guler et. al., reported that a single-dose bolus injection of dexmedetomidine before tracheal extubation attenuates airway reflexes measured by a coughing score [29]. Our finding that dexmedetomidine attenuates efferent C-fiber mediated contraction would provide a possible mechanistic basis for this clinical observation.

The clinically recommended intravenous infusions of dexmedetomidine are in the range of 0.2 – 0.7 μg/kg/h to achieve plasma concentrations of 0.3–2.5 ng/ml [37]. 2.5 ng/ml corresponds to approximately 12 nM. Although the effective concentrations in the external buffer in organ bath studies were higher than those reported in plasma during clinical use, it is difficult to make direct comparisons between exogenous buffer concentrations in an organ bath and plasma concentrations in vivo. Therefore, we measured tracheal tissue dexmedetomidine concentrations after tracheal rings were incubated in the buffer containing various buffer concentrations of dexmedetomidine, and found that the tissue concentrations (0.54–26 nM) were within the range of plasma concentrations achieved in clinical use.

At least four types of innervation are present in the airways including cholinergic nerves that release acetylcholine, adrenergic sympathetic nerves that release norepinephrine and excitatory or inhibitory nonadrenergic noncholinergic nerves (C-fibers) which release substance P, vasoactive intestinal polypeptide (VIP), calcitonin gene-related peptide (CGRP) and nitric oxide. In guinea pig trachea, an ultrastructural study revealed close proximity of adrenergic and cholinergic nerve varicosities [38] suggesting presynaptic modulation of acetylcholine release by the adrenergic system. Indeed, presynaptic adrenergic inhibition of cholinergic neurotransmission in cardiac vagal nerve terminals [39] was reported. Therefore, we questioned whether effects of dexmedetomidine on cholinergic EFS-induced contractions could be mediated by dexmedetomidine acting upon sympathetic nerves which in turn modulated parasympathetic cholinergic nerves or directly on β2 adrenoceptors on airway smooth muscle to induce relaxation. Virtanen et al. reported in receptor binding experiments and isolated organs that medetomidine was devoid of affinity or effects on β1 or β2 adrenoceptors [9], but the highest concentration of medetomidine evaluated in those experiments was 10 μM. To exclude the possibility that in the current study, 100 μM of dexmedetomidine was activating β2 adrenoceptors on cholinergic nerves or airway smooth muscle, 1 μM propranolol (non-selective β-adrenoceptor antagonist) was included in the organ bath buffer. Even in the presence of propranolol, cholinergic EFS-induced contraction was reduced by dexmedetomidine (data not shown).

Remifentanil is another agent frequently used during awake intubation or extubation of the trachea that has analgesic and antitussive properties. Previous clinical studies comparing dexmedetomidine and remifentanil demonstrated relatively similar awake intubation and post-intubation conditions, with similar efficacies for sedation levels, hemodynamic profiles and the success rates of intubation [28, 40]. The advantages of remifentanil include its ultra-short duration of action with a consistent half-life, suppression of coughing and attenuation of cardiovascular responses during airway manipulation, but with some disadvantages including oxygen desaturation and a higher incidence of awareness during intubation. Previously, EFS-induced cholinergic responses were reported to be inhibited by a μ-opioid receptor agonist in guinea pig [41] and human [6] airways in EFS-stimulus frequency-dependent manner, but in these previous studies direct measurements of exogenous acetylcholine-induced contractions or neural acetylcholine release were not performed. In the present study, remifentanil did not inhibit 30Hz EFS-induced contraction which is consistent with previous reports using a selective μ-opioid receptor agonist [6, 42]. In contrast, remifentanil did attenuate a 2Hz EFS-induced contraction, and the attenuation was reversed by the opioid receptor antagonist naloxone (data not shown). However, we did not detect a reduction in the amount of acetylcholine released at 2Hz by remifentanil indicating that the attenuation of contractile force at 2Hz is μ-opioid receptor mediated but not due to decreased acetylcholine release. It is possible that the inhibition of EFS-induced contractions by opioid agonists is due to increase in the release of inhibitory neurotransmitters from nonadrenergic noncholinergic nerves, however, testing all possible neurotransmitters released is beyond the scope of current study. In the central nervous system, direct measurement of acetylcholine release in the pontine brain stem in cats showed decreased release by fentanyl and morphine, but not by remifentanil [43]. Our study showed that remifentanil did not attenuate 2Hz EFS-induced acetylcholine release consistent with this previous study finding. Additionally, remifentanil had no effect on direct airway smooth muscle contraction induced by exogenously applied acetylcholine. Taken together these findings suggest that remifentanil can relax a 2Hz but not 30Hz EFS-induced contraction mediated through a μ-opioid receptor but that this relaxation is not due to a reduction in acetylcholine release. It has been reported that either intravenous or inhaled lidocaine can attenuate reflex-induced bronchoconstriction in humans [44] via direct effects on smooth muscle cells [45] by decreasing intracellular calcium [46] and via neural blockade of vagal reflex or cough pathways [47]. Thus, lidocaine is also frequently used as an adjunct to prevent reflex-induced bronchoconstriction during airway manipulation. Lidocaine has also been reported to relax acetylcholine-induced contraction in rat trachea [48]. In fact, clinical studies comparing dexmedetomidine and remifentanil [28, 40] also included clinically relevant doses of lidocaine, such that potential lidocaine effects can’t be easily distinguished from the effects of the study drugs. Our results suggests that lidocaine’s effect on airway are by inhibition of afferent C-fibers and direct muscle relaxation, and not by inhibition of acetylcholine release from cholinergic nerves. Tetrodotoxin, which is known to abolish cholinergic contractions by blockade of sodium channels [24], decreased acetylcholine release while lidocaine did not. Lidocaine, on the other hand, attenuated afferent C-fiber mediated contraction while tetrodotoxin did not. In the sensory peripheral nervous system, tetrodotoxin-resistant voltage-gated sodium channels NaV 1.8 and NaV 1.9, which are found mostly in small dorsal root ganglion cells associated with thin fibers, play an important role in impulse generation, and the IC_50_ for lidocaine blockade of these impulses was 177 +/− 25 μM [49]. These previous findings are consistent with the present findings which show an effect of lidocaine on capsaicin-induced contraction and a lack of effect by tetrodotoxin.

## Conclusions

Our findings suggest that dexmedetomidine, but not lidocaine or remifentanil attenuates acetylcholine release during cholinergic EFS in the airway and may provide a plausible mechanism for the observed utility of dexmedetomidine in attenuating airway reactivity during airway manipulation, and in facilitating noninvasive ventilation in critically ill asthmatics [50] where dysregulated acetylcholine release is suggested. Dexmedetomidine also attenuates C-fiber mediated contraction, which may be an underlying mechanism for cough suppression by dexmedetomidine. With its known clinical advantages of establishing a comfortable, calm patient who is arousable and cooperative [51] without respiratory depression, dexmedetomidine makes an excellent drug of choice during awake airway instrumentation or during emergence from general anesthesia when smooth extubation is desired, and for patients with obstructive airway disease, who are difficult to wean from mechanical ventilation.

## Abbreviations

AVPN: Airway-related vagal preganglionic neurons
CGRP: Calcitonin gene-related peptide
COPD: Chronic obstructive pulmonary disease
EFS: Electrical field stimulation
GABA: γ-aminobutyric acid
MS: Mass spectrometry
PBS: Phosphate buffered saline
TRPV1: Transient receptor potential cation channel subfamily V member 1
VIP: Vasoactive intestinal polypeptide
NANC: non-adrenergic non-cholinergic

## References

[1] Irwin RS (2006). Complications of cough: ACCP evidence-based clinical practice guidelines. Chest.

[2] Heunks LM, van der Hoeven JG (2010). Clinical review: the ABC of weaning failure--a structured approach. Crit Care.

[3] Canning BJ, Woo A, Mazzone SB (2012). Neuronal modulation of airway and vascular tone and their influence on nonspecific airways responsiveness in asthma. J Allergy.

[4] Larsen GL, Fame TM, Renz H (1994). Increased acetylcholine release in tracheas from allergen-exposed IgE-immune mice. Am J Physiol.

[5] van der Velden VH, Hulsmann AR (1999). Autonomic innervation of human airways: structure, function, and pathophysiology in asthma. Neuroimmunomodulation.

[6] Belvisi MG, Stretton CD, Verleden GM, Ledingham SJ, Yacoub MH, Barnes PJ (1992). Inhibition of cholinergic neurotransmission in human airways by opioids. J Appl Physiol.

[7] Bowery NG (1993). GABAB receptor pharmacology. Annu Rev Pharmacol Toxicol.

[8] Langer SZ (1980). Presynaptic regulation of the release of catecholamines. Pharmacol Rev.

[9] Virtanen R, Savola JM, Saano V, Nyman L (1988). Characterization of the selectivity, specificity and potency of medetomidine as an alpha 2-adrenoceptor agonist. Eur J Pharmacol.

[10] Virtanen R (1986). Antinociceptive activity and mechanism of action of detomidine. J Vet Pharmacol Ther.

[11] Doze VA, Chen BX, Maze M (1989). Dexmedetomidine produces a hypnotic-anesthetic action in rats via activation of central alpha-2 adrenoceptors. Anesthesiology.

[12] Kauppila T, Kemppainen P, Tanila H, Pertovaara A (1991). Effect of systemic medetomidine, an alpha 2 adrenoceptor agonist, on experimental pain in humans. Anesthesiology.

[13] Venn RM, Bradshaw CJ, Spencer R (1999). Preliminary UK experience of dexmedetomidine, a novel agent for postoperative sedation in the intensive care unit. Anaesthesia.

[14] Belleville JP, Ward DS, Bloor BC (1992). Maze M Effects of intravenous dexmedetomidine in humans. I. Sedation, ventilation, and metabolic rate. Anesthesiology.

[15] Venn RM, Hell J, Grounds RM (2000). Respiratory effects of dexmedetomidine in the surgical patient requiring intensive care. Crit Care.

[16] Gurbet A, Basagan-Mogol E, Turker G, Ugun F, Kaya FN, Ozcan B (2006). Intraoperative infusion of dexmedetomidine reduces perioperative analgesic requirements. Can J Anaesth.

[17] Scheinin B, Lindgren L, Randell T, Scheinin H, Scheinin M (1992). Dexmedetomidine attenuates sympathoadrenal responses to tracheal intubation and reduces the need for thiopentone and peroperative fentanyl. Br J Anaesth.

[18] Unger RJ, Gallagher CJ (2006). Dexmedetomidine sedation for awake fiberoptic intubation. Seminars in Anesthesia, Perioperative Medicine and pain.

[19] Jooste E, Zhang Y, Emala CW (2005). Rapacuronium preferentially antagonizes the function of M2 versus M3 muscarinic receptors in guinea pig airway smooth muscle. Anesthesiology.

[20] Baker DG, Don HF, Brown JK (1992). Direct measurement of acetylcholine release in guinea pig trachea. Am J Physiol.

[21] Rhoden KJ, Meldrum LA, Barnes PJ (1988). Inhibition of cholinergic neurotransmission in human airways by beta 2-adrenoceptors. J Appl Physiol.

[22] Grundstrom N, Andersson RG, Wikberg JE (1981). Prejunctional alpha 2 adrenoceptors inhibit contraction of tracheal smooth muscle by inhibiting cholinergic neurotransmission. Life Sci.

[23] Yu M, Wang Z, Robinson NE (1993). Prejunctional alpha 2-adrenoceptors inhibit acetylcholine release from cholinergic nerves in equine airways. Am J Physiol.

[24] Ellis JL, Undem BJ (1990). Non-adrenergic, non-cholinergic contractions in the electrically field stimulated guinea-pig trachea. Br J Pharmacol.

[25] Lee LY, Pisarri TE (2001). Afferent properties and reflex functions of bronchopulmonary C-fibers. Respir Physiol.

[26] Karlsson JA, Sant’Ambrogio G, Widdicombe J (1988). Afferent neural pathways in cough and reflex bronchoconstriction. J Appl Physiol.

[27] Lodenius A, Ebberyd A, Hardemark Cedborg A (2016). Sedation with Dexmedetomidine or Propofol Impairs Hypoxic Control of Breathing in Healthy Male Volunteers: A Nonblinded, Randomized Crossover Study. Anesthesiology.

[28] Hu R, Liu JX, Jiang H (2013). Dexmedetomidine versus remifentanil sedation during awake fiberoptic nasotracheal intubation: a double-blinded randomized controlled trial. J Anesth.

[29] Guler G, Akin A, Tosun Z, Eskitascoglu E, Mizrak A, Boyaci A (2005). Single-dose dexmedetomidine attenuates airway and circulatory reflexes during extubation. Acta Anaesthesiol Scand.

[30] Aksu R, Akin A, Bicer C, Esmaoglu A, Tosun Z, Boyaci A (2009). Comparison of the effects of dexmedetomidine versus fentanyl on airway reflexes and hemodynamic responses to tracheal extubation during rhinoplasty: A double-blind, randomized, controlled study. Curr Ther Res Clin Exp.

[31] Senoglu N, Oksuz H, Dogan Z, Yildiz H, Kamaz A, Ugur N (2009). Effects of Dexmedetomidine on respiratory mechanics during mechanical ventilation. J Anaesth Clin Pharmacol.

[32] Groeben H, Mitzner W, Brown RH (2004). Effects of the alpha2-adrenoceptor agonist dexmedetomidine on bronchoconstriction in dogs. Anesthesiology.

[33] Yamakage M, Iwasaki S, Satoh JI, Namiki A (2008). Inhibitory effects of the alpha-2 adrenergic agonists clonidine and dexmedetomidine on enhanced airway tone in ovalbumin-sensitized guinea pigs. Eur J Anaesthesiol.

[34] Chang HC, Cherng YG, Hsu CT, Liu MC, Wang HW (2013). Effects of dexmedetomidine on the isolated rat tracheal smooth muscle. J Exp Clin Med.

[35] Thompson DC, Diamond L, Altiere RJ (1990). Presynaptic alpha adrenoceptor modulation of neurally mediated cholinergic excitatory and nonadrenergic noncholinergic inhibitory responses in guinea pig trachea. J Pharmacol Exp Ther.

[36] Kim JG, Sung HJ, Ok SH, et al. Calcium sensitization involved in dexmedetomidine-induced contraction of isolated rat aorta. Can J Physiol Pharmacol. 2011. 10.1139/y11-065 21861649

[37] Fujita Y, Inoue K, Sakamoto T (2013). A comparison between dosage and plasma concentrations of dexmedetomidine in clinically ill patients: a prospective, observational, cohort study in Japan. J Intensive Care.

[38] Jones TR, Kannan MS, Daniel EE (1980). Ultrastructural study of guinea pig tracheal smooth muscle and its innervation. Can J Physiol Pharmacol.

[39] Akiyama T, Yamazaki T (2000). Adrenergic inhibition of endogenous acetylcholine release on postganglionic cardiac vagal nerve terminals. Cardiovasc Res.

[40] Cattano D, Lam NC, Ferrario L (2012). Dexmedetomidine versus Remifentanil for Sedation during Awake Fiberoptic Intubation. Anesthesiol Res Pract.

[41] Belvisi MG, Stretton CD, Barnes PJ (1990). Modulation of cholinergic neurotransmission in guinea-pig airways by opioids. Br J Pharmacol.

[42] Pype JL, Dupont LJ, Demedts MG, Verleden GM (1996). Opioids modulate the cholinergic contraction but not the nonadrenergic relaxation in guinea-pig airways in vitro. Eur Respir J.

[43] Mortazavi S, Thompson J, Baghdoyan HA, Lydic R (1999). Fentanyl and morphine, but not remifentanil, inhibit acetylcholine release in pontine regions modulating arousal. Anesthesiology.

[44] Groeben H, Silvanus MT, Beste M, Peters J (1999). Both intravenous and inhaled lidocaine attenuate reflex bronchoconstriction but at different plasma concentrations. Am J Respir Crit Care Med.

[45] Okumura F, Denborough MA (1980). Effects of anaesthetics on guineapig tracheal smooth muscle. Br J Anaesth.

[46] Kai T, Nishimura J, Kobayashi S, Takahashi S, Yoshitake J, Kanaide H (1993). Effects of lidocaine on intracellular Ca2+ and tension in airway smooth muscle. Anesthesiology.

[47] Nishino T, Hiraga K, Sugimori K (1990). Effects of i.v. lignocaine on airway reflexes elicited by irritation of the tracheal mucosa in humans anaesthetized with enflurane. Br J Anaesth.

[48] Lautner RQ, Zapata-Sudo G, Sudo RT (2009). Relaxation of tracheal smooth muscle independent on functional epithelium cells induced by lidocaine, bupivacaine and isomers in rats. Eur J Pharmacol.

[49] Poyraz D, Brau ME, Wotka F (2003). Lidocaine and octanol have different modes of action at tetrodotoxin-resistant Na (+) channels of peripheral nerves. Anesth Analg.

[50] Takasaki Y, Kido T, Semba K (2009). Dexmedetomidine facilitates induction of noninvasive positive pressure ventilation for acute respiratory failure in patients with severe asthma. J Anesth.

[51] Hall JE, Uhrich TD, Barney JA, Arain SR, Ebert TJ (2000). Sedative, amnestic, and analgesic properties of small-dose dexmedetomidine infusions. Anesth Analg.

